# Terpenes and Terpenoids Conjugated with BODIPYs: An Overview of Biological and Chemical Properties

**DOI:** 10.1021/acs.jnatprod.3c00961

**Published:** 2024-03-14

**Authors:** Jarmila Stanková, Michal Jurášek, Marián Hajdúch, Petr Džubák

**Affiliations:** † Institute of Molecular and Translational Medicine, Faculty of Medicine and Dentistry, Palacký University, 77900 Olomouc, Czech Republic; ‡ Laboratory of Experimental Medicine, Institute of Molecular and Translational Medicine, University Hospital Olomouc, 77900 Olomouc, Czech Republic; § Department of Chemistry of Natural Compounds, University of Chemistry and Technology Prague, 16628 Prague, Czech Republic

## Abstract

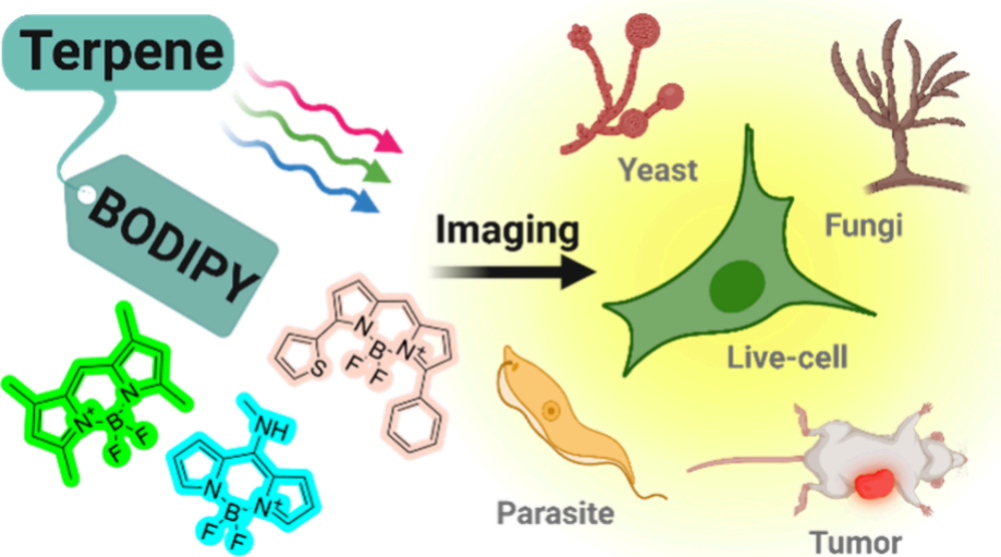

Advancements in small-molecule research have created the need for sensitive techniques to accurately study biological processes in living systems. Fluorescent-labeled probes have become indispensable tools, particularly those that use boron–dipyrromethene (BODIPY) dyes. Terpenes and terpenoids are organic compounds found in nature that offer diverse biological activities, and BODIPY-based probes play a crucial role in studying these compounds. Monoterpene–BODIPY conjugates have exhibited potential for staining bacterial and fungal cells. Sesquiterpene–BODIPY derivatives have been used to study sarcoplasmic/endoplasmic reticulum calcium ATPase (SERCA), indicating their potential for drug development. Owing to their unique properties, diterpenes have been investigated using BODIPY conjugates to evaluate their mechanisms of action. Triterpene–BODIPY conjugates have been synthesized for biological studies, with different spacers affecting their cytotoxicity. Fluorescent probes, inspired by terpenoid-containing vitamins, have also been developed. Derivatives of tocopherol, coenzyme Q10, and vitamin K1 can provide insights into their oxidation–reduction abilities. All these probes have diverse applications, including the study of cell membranes to investigate immune responses and antioxidant properties. Further research in this field can help better understand and use terpenes and terpenoids in various biological contexts.

## Introduction

In recent years, considerable advancements have been made in identifying the mechanisms of action (MOA) and cellular targets of small molecules. However, current methods often rely on indirect assays or involve cell lysis, which may not accurately reflect biological processes in living systems, such as live cells, tissues, or animals.^1^ To effectively identify target proteins and conduct MOA studies, developing strategies that can sensitively detect biological processes in living systems are necessary, such as live-cell microscopy.^2^ Fluorescent-labeled probes of bioactive small molecules have been used for a long time^3,4^ and have emerged as powerful tools for exploring biological phenomena and the MOA of these molecules.^5−7^ This approach allows for drug uptake and subcellular distribution imaging.^7^ In addition to the direct tracking of distribution, another method involves using probes for membrane trafficking.^8^ Moreover, in-gel fluorescent band visualization following sodium dodecyl–sulfate polyacrylamide gel electrophoresis separation can be used to visualize the drug–protein target complex, even under denaturing conditions.^9^

In general, a bioconjugate comprises a luminophore conjugated with a drug or another biological compound via a linker. The luminophore can serve as a drug by actively participating in cancer cell destruction and acting as a photosensitizer in photodynamic therapy.^10^ Boron–dipyrromethene (BODIPY) dyes represent ideal luminophores. Key features of these dyes include their intense and vibrant colors, high molar absorptivity, and fluorescence emission across a broad range of wavelengths. These properties render them ideal for fluorescence-based techniques, such as fluorescence microscopy, flow cytometry, and bioimaging. By implementing straightforward structural modifications, the crucial characteristics of BODIPY derivatives can be regulated with less effort compared to other dyes.^11^ The developed BODIPY bioconjugates exhibit distinct properties, particularly in the context of medical and biochemical research. The specific characteristics of each conjugate are determined by the nature of its components and the conjugation method used.^10^ Additionally, owing to their lipophilic nature, BODIPYs are suitable for labeling lipophilic substances. This is particularly relevant because the lipophilic character of biologically active compounds, such as terpenes, is often essential for their original action in living systems.

Terpenes and terpenoids are a broad and diverse group of organic compounds found in plants and other organisms. The repeating isoprene units comprise several structures and functional groups that contribute to their wide range of biological activities.^12^ For example, some of these natural compounds have anti-inflammatory properties,^13,14^ whereas others have antifungal,^15,16^ antibacterial,^17^ antiviral,^18,19^ and antitumor^20^ activities.

Several applications of BODIPY-based probes have been widely discussed in detail.^21^ In 2022, Antina et al.^10^ provided an overview of the BODIPY conjugates used in medical diagnostics and treatment. Herein, a unique examination of BODIPY conjugates in terpene and terpenoid research is provided, highlighting their significant impact on the elucidation of the biological activities of these compounds. We investigate the role of BODIPY-labeled terpene conjugates, emphasizing their contribution to advancing research across a spectrum of biological applications in contemporary research.

## Monoterpenes and Monoterpenoids

**Figure 1:**
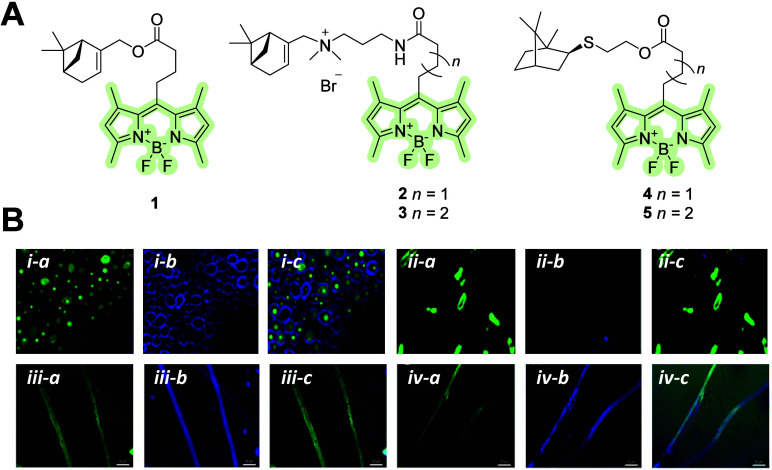
(A) Molecular structures of monoterpene–BODIPY conjugates **1**–**5**. (B) Fluorescence microscopy images of myrtenol–BODIPY ester **1** in *Candida albicans* (B-*i*) and *Escherichia coli* (B-*ii*).^23^ Copyright 2021, American Chemical Society. Visualization of myrtenol–BODIPY ester **1** (B-*iii*) and the cationic myrtenol–BODIPY **2** in filamentous fungi (*F. solani*) (B-*iv*).^24^ Copyright 2023, Guseva et al., Licensee MDPI, Basel, Switzerland. BODIPY–monoterpene conjugate (B*i*-*a*, *ii*-*a*, *iii*-*a*, and *iv*-*a*); calcofluor-white (B*i*-*b*, *ii*-*b*, *iii*-*b*, and *iv*-*b*); and overlay (B*i*-*c*, *ii*-*c*, *iii*-*c*, and *iv***-**c).

Monoterpenes comprise two isoprene units and are known for their strong and characteristic aroma. They are commonly found in various plants, including fruits, herbs, and essential oils, where they play important roles in defense mechanisms against herbivores and pathogens and aid in attracting pollinators. Several applications of monoterpenes conjugated with *meso*-modified BODIPYs were reported by Guseva et al. (Figure 1A).^22−26^ Changes in the BODIPY structure have been suggested to improve the properties of the dye, such as photostability and affinity of the molecule to targets. The *meso*-substituted BODIPY stains Gram-positive bacteria and can thus be used for the differential staining of Gram-positive and -negative bacteria in mixed cultures.^22^ To increase the efficiency of penetration into bacterial and fungal cells, the *meso*-substituted BODIPY was combined with myrtenol–bicyclic alcohol monoterpenes that possessed anti-inflammatory, antinociceptive, and antifungal activities.^27,28^ Myrtenol–BODIPY conjugate **1** is an uncharged, lipophilic aromatic compound that tends to penetrate bacterial, mammalian, and fungal cells rapidly and binds to membranes (Figure 1B).^23^ Furthermore, Guseva et al.^24^ introduced a quaternary ammonium fragment into the structure and synthesized myrtenol–BODIPY conjugates **2** and **3** with spacers containing three or four CH_2_ groups, respectively. The tested myrtenol–BODIPY **2** was localized in fungal cells (Figure 1B) and was also able to stain bacteria. A thioterpene moiety was introduced at the *meso*-position of BODIPY in subsequent studies.^25,26^ The thioterpene–BODIPY conjugate **4** actively penetrates erythrocytes and is poorly removed, even after washing with phosphate-buffered saline. This property ensures nontoxic staining of erythrocytes.^25^ Furthermore, thioterpene–BODIPY **5** exhibits considerable antiplatelet and anticoagulant activities. Molecular docking revealed that conjugating BODIPY with thioterpenoids enhanced dye affinity for the platelet receptor P2Y12.^26^ The combination of tailored BODIPY properties and natural properties of monoterpene/monoterpenoid exhibited promising results in the field of fluorescent probe development.

## Sesquiterpenes

**Figure 2:**
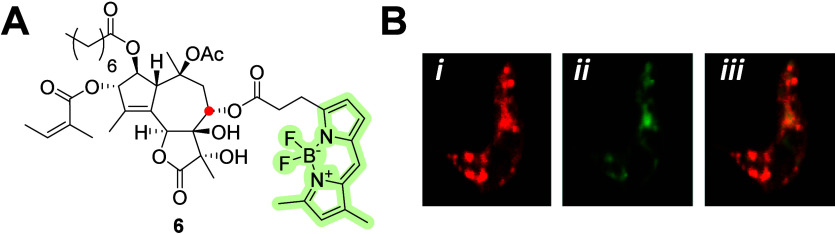
(A) Molecular structure of BODIPY–thapsigargin derivative **6** with a fluorophore attached at the C-8 (red dot) position. Fluorescent images of *T. evansi* parasite stained with H-300 anti-SERCA antibody (B-*i*), BODIPY-FL thapsigargin **6** (B-*ii*), and merged (B-*iii*).^36^ Copyright 2015, Elsevier Ltd.

Sesquiterpenes are composed of three isoprene units and often contribute to the distinct aromas of plants and fungi. Several sesquiterpenes have been studied for their potential medicinal properties. For example, artemisinin, a sesquiterpene lactone derived from sweet wormwood plants, is a critical element in the treatment of malaria.^29^ Other sesquiterpenes have been investigated for their potential anticancer, anti-inflammatory, and immunomodulatory properties.^30^ Thapsigargin is a sesquiterpene that specifically inhibits sarcoplasmic/endoplasmic reticulum (ER) calcium ATPase (SERCA). The binding site of thapsigargin at SERCA was described by Skytte et al.,^31^ and the mechanism of inhibition in eukaryotic organisms was studied previously.^32^ The fluorescent commercial probe thapsigargin–BODIPY derivative **6** (Figure 2A) has widely contributed to SERCA research.^33−35^ Recently, Pérez-Gordones et al.^36^ used conjugate **6** to localize a SERCA-like calcium pump in *Trypanosoma evansi* (Figure 2B). These results showed that thapsigargin or thapsigargin derivatives could be used as anti-*T. evansi* drugs.

**Figure 3:**
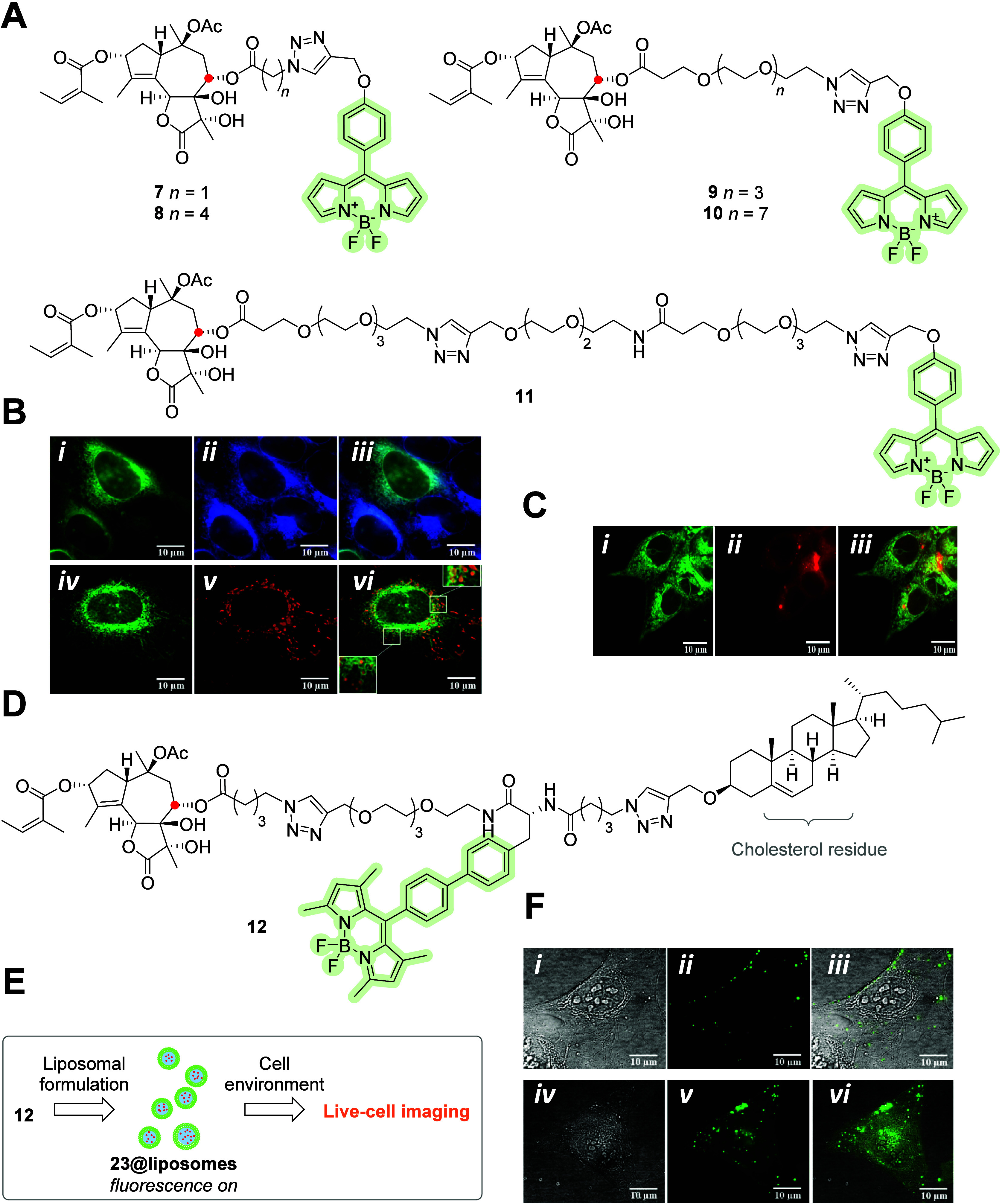
(A) Molecular structures of trilobolide–BODIPY **7**–**11** conjugated via an ester moiety at C-8 (red dot). (B) Live-cell images of **8** and **9** in U2OS cells. ER stained with **8** (B-*i*), ER-tracker blue-white DPX (B-*ii*), and overlay (B-*iii*). Live-cell images of **9** (B-*iv*), mitochondria stained with MitoTracker Red FM (B-*v*), and overlay (B-*vi*). (C) Visualization of *in situ* production of NO in MCF-7 cells via the DAR-2 probe: localization of **8** (C-*i*), activated DAR-2 probe (C-*ii*), and merged (C-*iii*).^37^ Copyright 2014, American Chemical Society. (D) Molecular structure of trilobolide–BODIPY **12** conjugated via the ester moiety with a PEG linker and cholesterol residue. (E) Formulation of construct **12** into **12**@liposomes. (F) Imaging of intracellular localization of **12**@liposomes in U2OS cells: bright-field images (F-*i* and F-*iv*), localization of **12**@liposomes images (F-*ii* and F-*v*), and merged images (F-*iii* and F-*vi*).^38^ Copyright 2017, Škorpilová et al., Licensee Beilstein-Institut.

Trilobolide, with a sesquiterpene lactone structure, is similar to thapsigargin and is assumed to interact with SERCA protein. Jurášek et al.^37^ synthesized and evaluated the biological activities of five fluorescent trilobolide–BODIPY conjugates, **7**–**11** (Figure 3A). The designed conjugates differed according to the nature of linkers and were labeled with green-emitting BODIPY. Only trilobolide–BODIPY **8** and **9** preserved the biological effect and were localized in the ER of several cell lines; see colocalization observed in the U2OS cell line in Figure 3B. Fragmentation of the mitochondrial network has also been reported, corresponding to the effects of trilobolide. Both trilobolide–BODIPY **8** and **9** induced nitric oxide release in cancer cell lines and primary immune cells and induced cytokine secretion in primary immune cells. Thus, both these probes can be used to further explore the molecular mechanism of trilobolide. Škorpilová et al.^38^ showed the synthesis of liposomes from trilobolide–BODIPY **12**. Figure 3D–F show the structure and localization of trilobolide–BODIPY **12** in U2OS cells. The liposomal construct was designed to increase the aqueous solubility of trilobolide. The fluorescent liposomes mainly stained lipid compartments in living cells and, therefore, do not seem to be promising for the delivery of SERCA inhibitors into cancer cells.

## Diterpenes and Diterpenoids

**Figure 4:**
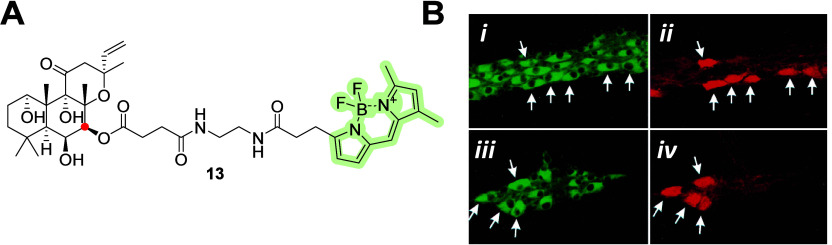
(A) Molecular structure of forskolin–BODIPY **13** with a fluorophore attached at the C-7 (red dot) position via an ester bond. (B) Fluorescence colabeling microscopy images of myenteric ganglia with **13** and calbindin-D28. Forskolin–BODIPY **13**-labeled neurons in the myenteric ganglion (B-*i*). The same ganglion was subjected to immunofluorescence labeling with primary antiserum to calbindin-D28, where the secondary antibody was labeled with Texas Red (B-*ii*). Another ganglion in a different tissue was labeled with **13** (B-*iii*). The same ganglion in (B-*iii*) showed colabeling with calbindin-D28 (B-*iv*).^39^ Copyright 1998, Springer-Verlag, Berlin, Heidelberg.

Diterpenes consist of four isoprene units and have diverse roles in nature, including serving as defense compounds against predators, contributing to the aroma of certain plants, and playing substantial roles in ecological interactions. Some diterpenes also have medicinal properties and are used in traditional medicine and modern pharmaceuticals. Diterpenes conjugated with BODIPY present a broad spectrum of compounds and applications. Many of these conjugates are commercially available and have been very popular, and some can be purchased for experiments. One of the labeled diterpenes is forskolin, conjugate **13** (Figure 4A), which has been used in several studies. For example, Liu et al.^39^ showed forskolin-BODIPY **13**’s usage as a fluorescent marker for membrane adenylyl cyclase in living enteric neurons in the guinea pig ileum. Forskolin–BODIPY **13** uses the activity of forskolin in adenylyl cyclase types I–VIII and is a suitable neural marker for identifying various classes of neurons. This was proved by colocalization experiments with specific calcium-binding proteins, such as calbindin-D28, as shown in Figure 4B.

**Figure 5:**
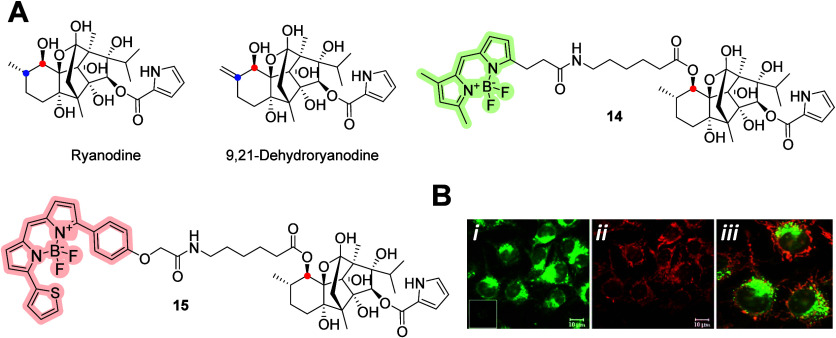
(A) Ryanodine-based BODIPY probes **14** and **15**, the mixtures of ryanodine and 9,21-dehydroryanodine, are conjugated with BODIPY-FL or BODIPY-TR-X via an ester bond, “probably” at the C-10 (red dot) position. MCF-7 cells were stained with BODIPY-FL-X thapsigargin (B-*i*), BODIPY-TR-X ryanodine **15** (B-*ii*), and merged (B-*iii*).^41^ Copyright 2008, Springer Science Business Media, LLC.

Fluorescent probes for the diterpene ryanodine have been reported previously. This alkaloid, originally used as an insecticide, has a high affinity for the ryanodine receptor (deriving its name) and can lock it in a half-open or closed state. In mammalian cells, this receptor is related to Ca^2+^ release from the sarcoplasmic reticulum and drives muscle contraction.^40^ Ryanodine–BODIPY **14** and **15** (Figure 5A) are commercially available, and conjugate **15** was used by Saldana et al.,^41^ who characterized the ryanodine receptor in MCF-7 cells. They applied two different fluorescent probes, BODIPY-TR-X ryanodine and BODIPY-FL-X thapsigargin, for the subcellular localization of the ryanodine receptor and evaluation of its colocalization with SERCA (Figure 5B) for staining MCF-7 cells. The ryanodine receptor was found in the perinuclear zone and adjacent to the endomembrane of cells and did not colocalize with thapsigargin-sensitive Ca^2+^-ATPase. Saldana et al. demonstrated that MCF-7 cells express ryanodine receptor type 1 by molecular cloning techniques.

**Figure 6:**
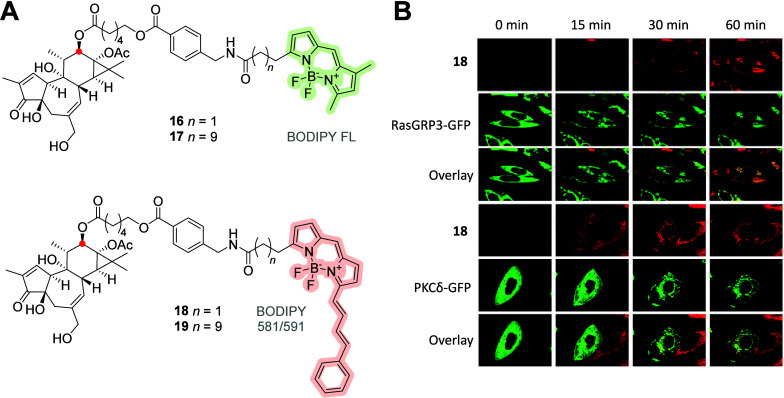
(A) Molecular structures of ester-based phorbol–BODIPY conjugates **16**–**19** attached at the C-10 (red dot) position. (B) Representative images of real-time visualization of red-emitting probe **18** with green fluorescent protein (GFP)-labeled RasGRP3 or PKCδ (green) in Chinese hamster ovary cells. Adapted from Braun, D. C., et al., 2005, with permission from AACR.^42^

Labeled diterpenes have been used to investigate the MOA of phorbol esters. Braun et al.^42^ reported various conjugates of phorbol esters linked to BODIPY-FL (green) and BODIPY 581/591 (red) (Figure 6A). These conjugates were intentionally designed with varying lipophilicities, resulting in different cellular uptake properties and affinity levels toward their natural targets, protein kinase C (PKC) and RasGRP. They act as tumor promoters via their interaction with PKC and RasGRP, and Braun et al. showed the colocalization and co-migration of receptors and ligands in real-time analysis;^43^ the images of phorbol–BODIPY **18** are shown in Figure 6B. A similar behavior of RasGRP was reported later.

**Figure 7:**
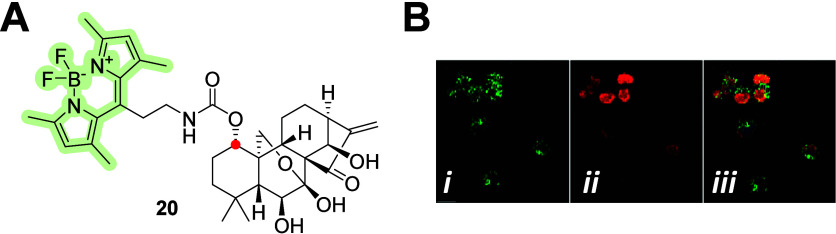
(A) Molecular structure of C-1-linked (red dot) carbamate-based oridonin–BODIPY conjugate **20**. (B) Representative fluorescent images in the Jurkat cells labeled with **20** (B-*i*), nucleolin (B-*ii*), and merged (B-*iii*).^47^ Copyright 2018, Vasaturo et al.

The cytotoxic activity of the diterpene oridonin has been studied in various human cells, and its molecular target was identified as protein kinase B.^44,45^ In addition, the inhibition of NLRP3 by oridonin and its anti-inflammasome activity have been reported.^46^ Vasaturo et al.^47^ prepared a fluorescent derivative of oridonin, using BODIPY-FL as a fluorescent label, to study the mechanism of action, efficiency, and kinetics of oridonin uptake in leukemia-derived Jurkat cells. The oridonin–BODIPY conjugate **20** (Figure 7A) exhibited maximum fluorescence in cells after 2 h of exposure and possibly colocalized with nucleolin (images in Figure 7B), suggesting a direct interaction, which was confirmed in the following experiments.

**Figure 8:**
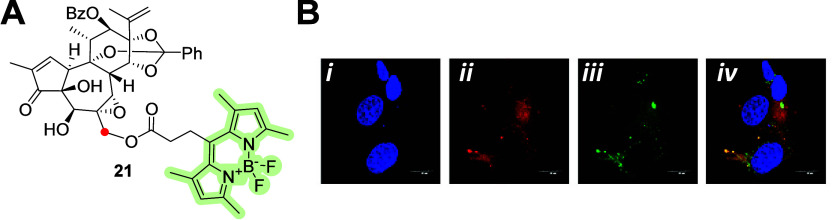
(A) Molecular structure of the C-20 (red dot) ester-linked daphnane–BODIPY conjugate **21**. (B) Representative fluorescent images in the **C4**–**2B** cells labeled with 4′,6-diamidino-2-phenylindole (DAPI) (B-*i*), anti-importin-β1 antibody (B-*ii*), **21** (B-*iii*), and merged (**B**-*iv*).^48^ Copyright 2022, American Chemical Society.

Another example of a diterpene with cytotoxic activity is a daphnane diterpene. A comprehensive study of its effects on castration-resistant prostate cancer (CRPC), including the use of daphnane–BODIPY conjugate **21**, was reported by Huang et al.^48^ in 2022. They showed selective growth inhibition of CRPC cells and complete blockage of tumor growth in preclinical models after treatment with a daphnane derivative containing benzoyl and phenyl groups and identified importin-β1 as its direct target. The colocalization of daphnane–BODIPY **21** and its structure are shown in Figure 8. Both these diterpenes, oridonin and the daphnane derivatives, are promising new molecules in the field of cancer treatment with new druggable targets, whereas paclitaxel represents a well-established drug in the treatment of breast and non-small-cell lung malignancies.^49^

**Figure 9:**
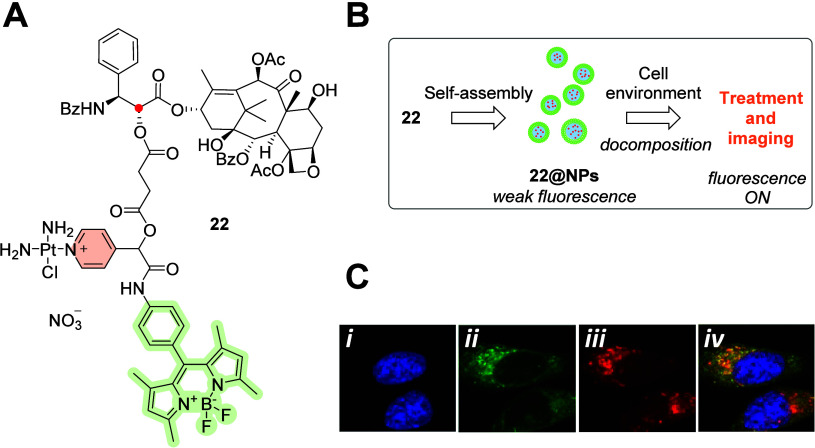
(A) Molecular structure of C-2 (red dot)-linked ester-based paclitaxel–Pt–BODIPY conjugate **22**. (B) Self-assembly of **22** into NPs. (C) Representative fluorescent images in A549 cells labeled with DAPI (C-*i*), **22@NPs** (C-*ii*), Lyso-Tracker Red (C-*iii*), and merged (C-*iv*).^52^ Copyright 2016, Wiley-VCH Verlag GmbH & Co. KGaA, Weinheim.

Paclitaxel causes mitotic arrest in tumor cells with microtubule stabilization by creating multiple hydrogen bonds within the β-tubulin-binding site.^50^ Its clinical limitations, such as solubility and toxicity, have promoted the development of taxoid analogs, including paclitaxel nanoparticles and BODIPY conjugates.^49^ Compared with free drugs, nanoparticles increase water solubility and accumulate preferentially in the tumor tissue. Additionally, fluorescent nanoparticles exhibit higher photostability and better biocompatibility than traditional fluorescent dyes. Similar to the use of drug carriers in the past, current approaches rely on supramolecular self-assembly in aqueous solutions.^51^ Sun et al.^52^ prepared amphiphilic paclitaxel–Pt–BODIPY conjugate **22** (Figure 9), in which a platinum moiety (Pt) was used as the hydrophilic head. Paclitaxel–Pt–BODIPY **22** can self-assemble into nanoparticles (**22@NPs**) in water. **22@NPs** penetrated human lung carcinoma (A549) cells via endocytosis, as shown in Figure 9C, and demonstrated high cytotoxicity against these cells and human breast cancer (MCF-7) cells.

**Figure 10:**
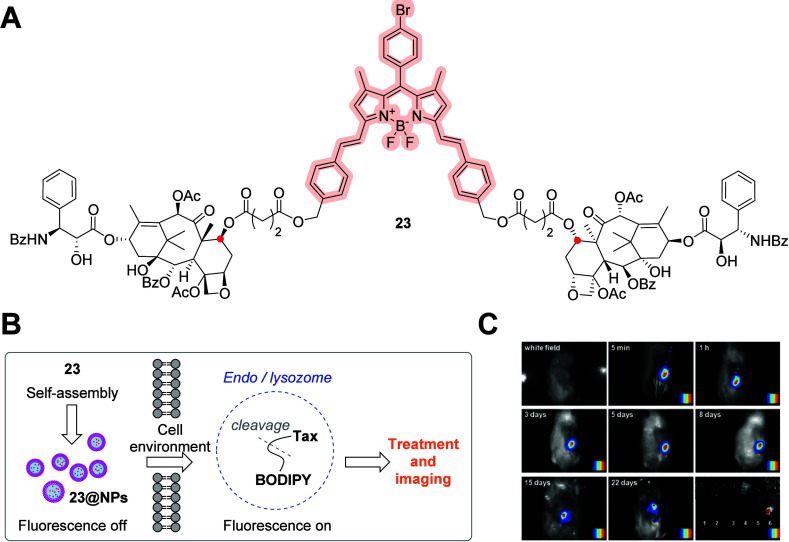
(A) Molecular structure of C-7 (red dot)-conjugated ester-based dimeric paclitaxel–NIR-BODIPY conjugate **23**. (B) Self-assembly of **23** into **23@NPs** and the proposed mechanism of action. (C) *In vivo* NIRF imaging of Kunming mice bearing U14 tumors after intratumorally injecting with **23@NPs**.^53^ Copyright 2018, Elsevier Ltd.

Furthermore, Zhang et al.^53^ reported the synthesis of paclitaxel–near-infrared (NIR)-BODIPY conjugate **23** (Figure 10A), which could self-assemble into spherical nanoparticles suitable for chemotherapy and bioimaging. **23@NPs** rapidly disassembled in the presence of proteinase K and accumulated in HeLa cell lysosomes, where various proteinases are naturally present. They exhibited potent cytotoxicity toward HeLa and HepG2 cells. *In vivo* experiments showed that **23@NPs** could be used for bioimaging without harming normal tissues (as seen in Figure 10C).^53^

**Figure 11:**
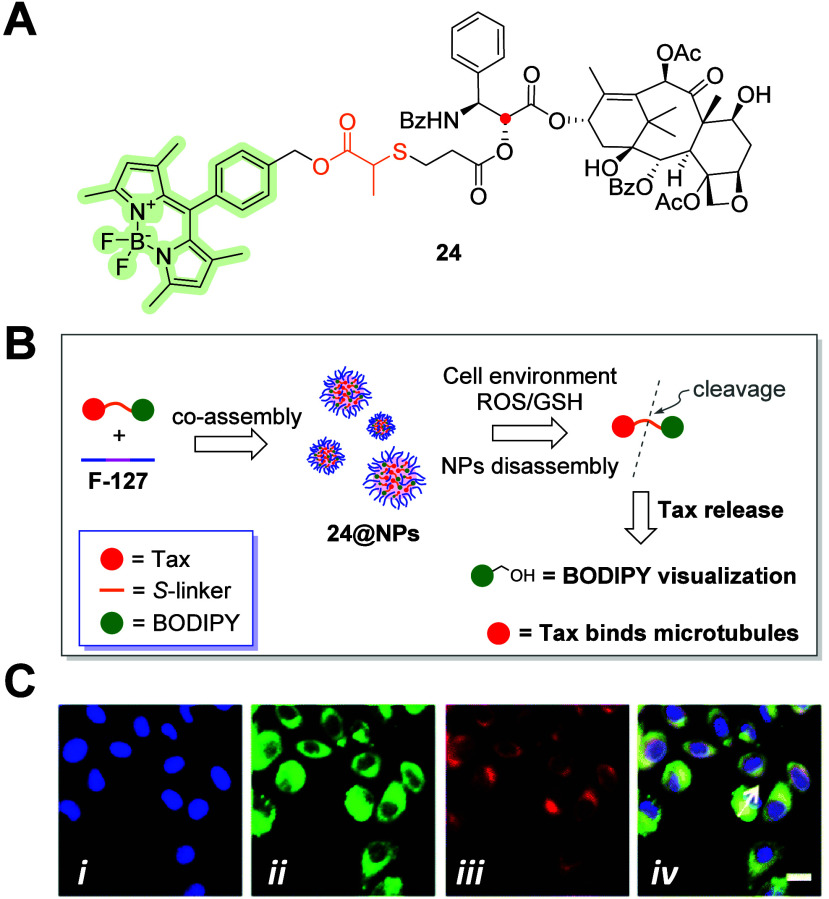
(A) Molecular structure of the C-2′ (red dot)-linked cleavable thioether moiety containing paclitaxel–BODIPY conjugate **24**. (B) Co-assembly of **24** into NPs and its proposed mechanism of action. (C) Representative fluorescent images in HeLa cells labeled with Hoechst (C-*i*), **24@NPs** (C-*ii*), Lyso-Tracker Red (C-*iii*), and merged (C-*iv*).^54^ Copyright 2021, Royal Chemical Society.

**Figure 12:**
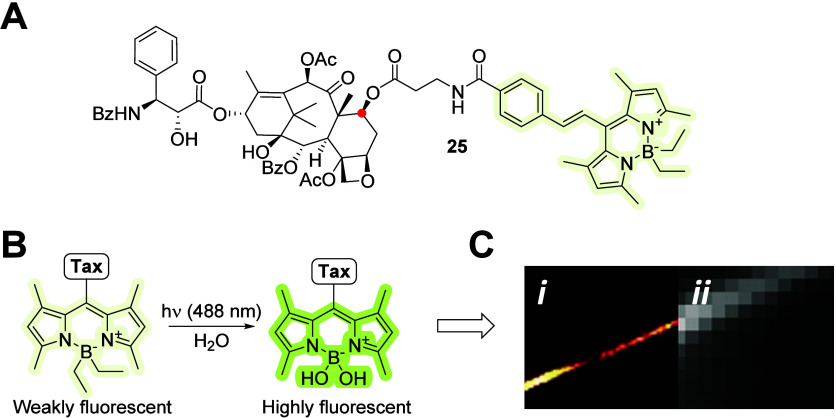
(A) Molecular structure of C-7 (red dot) ester-conjugated photoswitchable paclitaxel–BODIPY conjugate **25**. (B) Proposed mechanism of action; super-resolution image of an activated probe (C-*i*) and its diffraction-limited image (C-*ii*).^55^ Copyright 2018, Wiley-VCH Verlag GmbH & Co. KGaA, Weinheim.

Another activatable fluorescent prodrug of paclitaxel and BODIPY was prepared and characterized by Xia et al.^54^ Paclitaxel–BODIPY conjugate **24** (Figure 11A) contains a monosulfide linker that can be cleaved at high levels of glutathione or H_2_O_2_ in cancer cells. Controllable drug release and redox activation led to higher selectivity of prodrugs to cancer cells and reduced damage to normal cells.^54^ Various applications of paclitaxel-conjugated BODIPY have been reported by Wijesooriya et al.^55^ They synthesized a photoactivatable probe, paclitaxel–BODIPY conjugate **25** (Figure 12A), for localization-based super-resolution microscopy, where paclitaxel is responsible for targeting of microtubules. Paclitaxel–BODIPY conjugate **25** can be used to image live cells in various imaging buffers, even without the addition of reducing agents or oxygen scavengers. Photoactivation is accomplished using lower irradiation than alternative probes; thus, it is gentler for various biological samples that might otherwise be damaged by the high power or UV lasers.

## Triterpenes and Triterpenoids

Triterpenes comprise six isoprene units, making them larger than most other terpenes. Most have a characteristic structure consisting of several fused rings, which give them structural rigidity.^56^ One of the primary MOA of terpenes is their ability to modulate various cell signaling pathways. For example, triterpenes can inhibit the activity of certain enzymes involved in inflammation such as cyclooxygenase and lipoxygenase.^57^ They can also activate nuclear receptors such as peroxisome proliferator-activated receptor gamma,^58^ which are involved in regulating glucose and lipid metabolism. Another common mechanism is the downregulation of the NF-κB pathway and prevention of increased cytokine levels.^59^ Triterpenes can inhibit the multidrug resistance protein 1 (MDR1) efflux pump, which can be potentially used to improve the effectiveness of drugs.^60^ Compared with current anticancer drugs, the main advantage of triterpenes is that they can trigger apoptosis by an intrinsic pathway independent of DNA damage signaling,^61,62^ permeabilize the mitochondrial membrane, and release cytochrome *c* into the cytoplasm.^63^ Given their promising results in anticancer research, their MOA need to be clarified. The conjugation results of triterpene carboxylic acids, the triterpene betulin, and bevirimat derivatives with BODIPY dyes are described later. All these cases focus on the synthesis, supplemented by the basic biological characterization of the conjugates.

**Figure 13:**
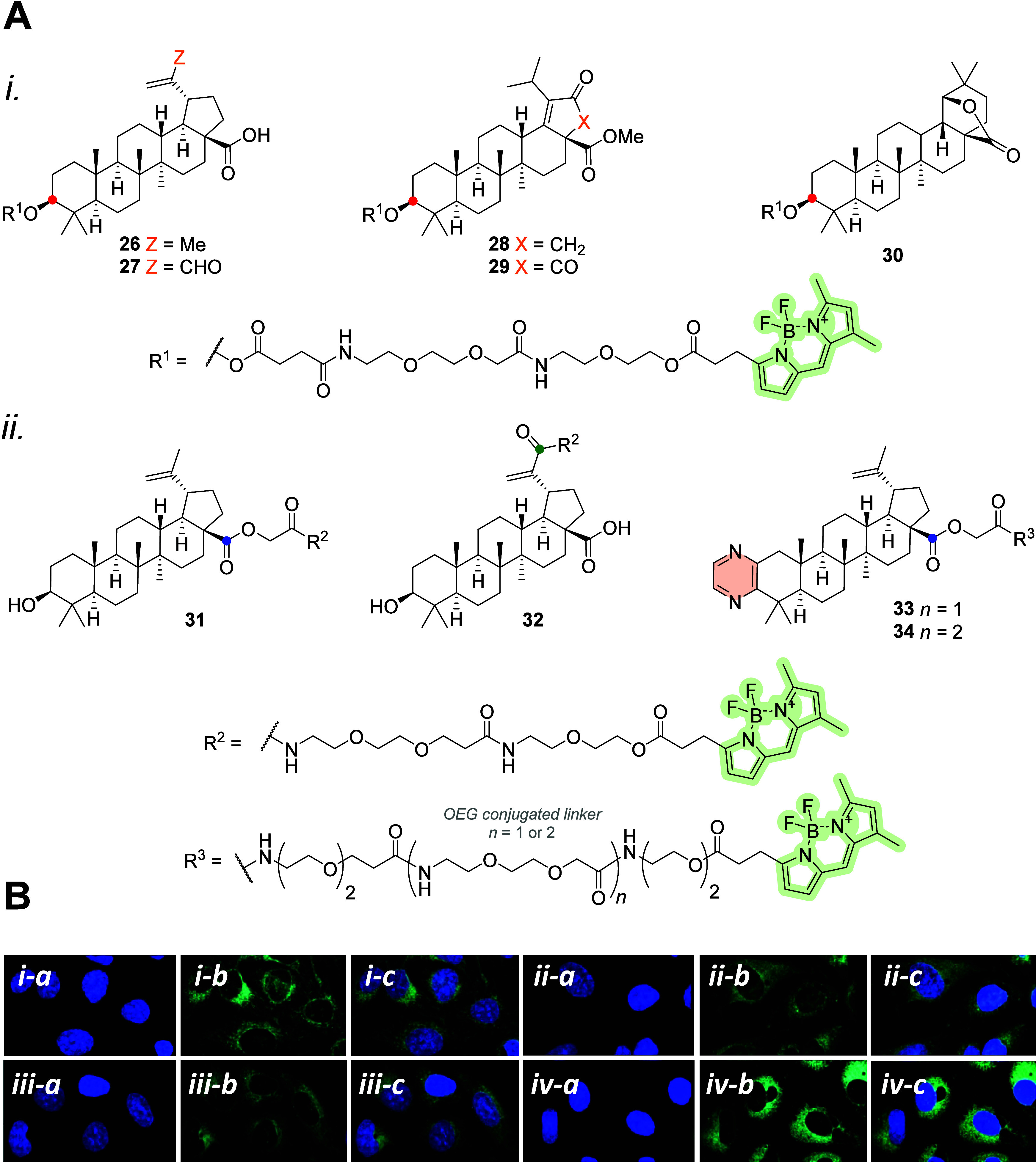
(A) Molecular structures of different triterpene–BODIPY **26**–**34** conjugated via an ester moiety at C-3 (red dot) (A-*i*) or C-28 (blue dot)/C-30 (green dot) (A-*ii*). (B) Live-cell localization of the cytotoxic probes in U2OS cells, namely, **26** (B-*i*), **27** (B-*ii*), **32** (B-*iii*), and **34** (B-*iv*). Staining with Hoechst (nuclei) (*i*–*iv*-*a*), localization of the triterpene-based BODIPY probes (*i*–*iv*-*b*), and merged (*i*–*iv*-*c*).^64^ Copyright 2018, Wiley-VCH Verlag GmbH & Co. KGaA, Weinheim.

The first study describing betulinic acid (BA) conjugation with BODIPY was performed by Krajcovicova et al. in 2018. They synthesized triterpene–BODIPY **26**–**34** and showed the results of linking the fluorescent BODIPY-FL tag at three different positions on the triterpene core: C-3 triterpene–BODIPY **26**, C-28 triterpene–BODIPY **31**, and C-30 triterpene–BODIPY **32** (Figure 13A). The conjugates of BA at positions C-28 and C-30 were almost inactive, suggesting that the pharmacophore is present in this part of the compound. In addition to the highly active derivative of BA with an aldehyde group at the C-30 position of triterpene–BODIPY **27**, only the conjugate of the triterpene with a fused pyrazine labeled at the C-28 position, triterpene–BODIPY **34**, was cytotoxic to various cell lines. Unlike triterpene–BODIPY **31**, the same triterpene structure (**34**, differing with a shorter linker to BODIPY-FL) was active only in the CCRF-CEM cell line. After short incubation periods, all conjugates were detected in living cells (Figure 13B), with possible colocalization in ER and mitochondria,^64^ which are known targets of BA.^65,66^

**Figure 14:**
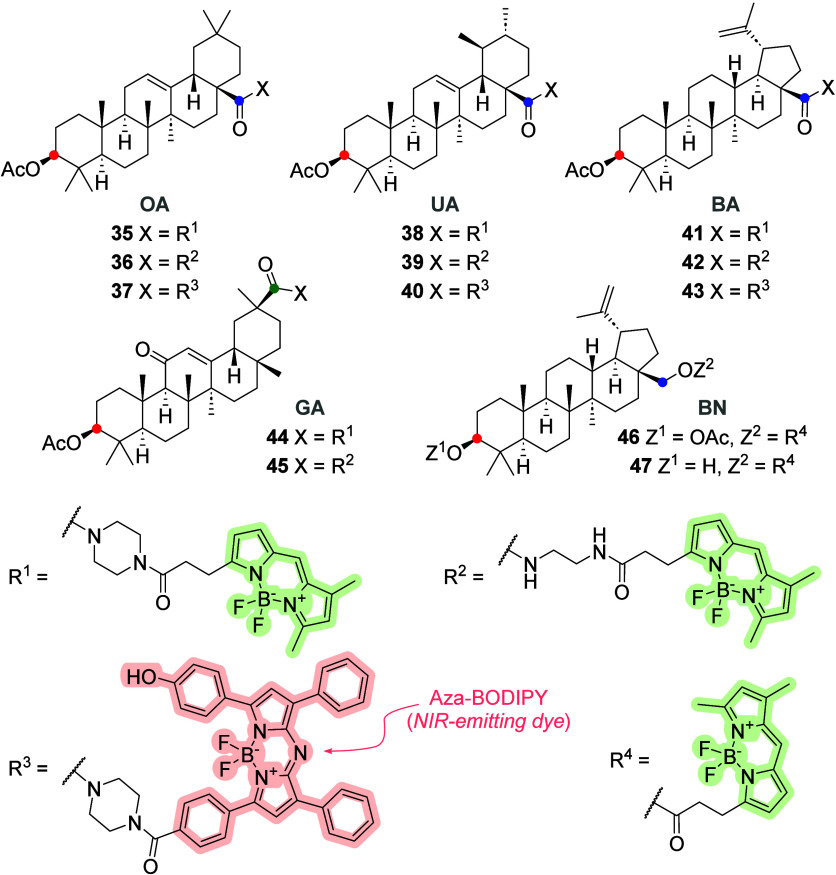
Molecular structures of different triterpene–BODIPY probes **35**–**47** (the acyl residues of OA, UA, BA, GA, and BA) conjugated via an amide or ester moiety at the C-3 (red dot), C-28 (blue dot), or C-30 (green dot) positions of the triterpene molecule.

Brandes et al.^67^ reported results of BA, oleanolic (OA), ursolic (UA), and glycyrrhetinic (GA) acids and betulin (BN) conjugated with BODIPY-FL; the structures of triterpene–BODIPY **35**–**37** are shown in Figure 14. They demonstrated the influence of different spacers between the terpenes and the BODIPY dyes. The best option was an ethylenediamine spacer in a conjugate of 3-*O*-acetylbetulinic acid with BODIPY-FL; the conjugate triterpene–BODIPY **42** was cytotoxic to MCF-7 cells but not to other cell lines. All ethylenediamine-derived amides were more cytotoxic than their piperazinyl-derived analogs. Only the 3-*O*-acetylated piperazinyl-derived amide of GA (triterpene–BODIPY **44**) was active in various cells, except HT-29. The difference in cytotoxicity depends on the chosen spacer and further proves the importance of a suitable spacer between the triterpene and the additionally introduced group.^67^ A similar result was described by Krajcovicova et al.^64^ by considering different spacer lengths. Later, they reported the synthesis and characterization of triterpenoids labeled with aza-BODIPY. The triterpenoid–aza-BODIPY conjugates **37**, **40**, and **43** did not exhibit cytotoxicity in various cell lines. Unfortunately, the localization of conjugates cannot be evaluated because microscopic images were poor in quality and resolution.^68^

**Figure 15:**
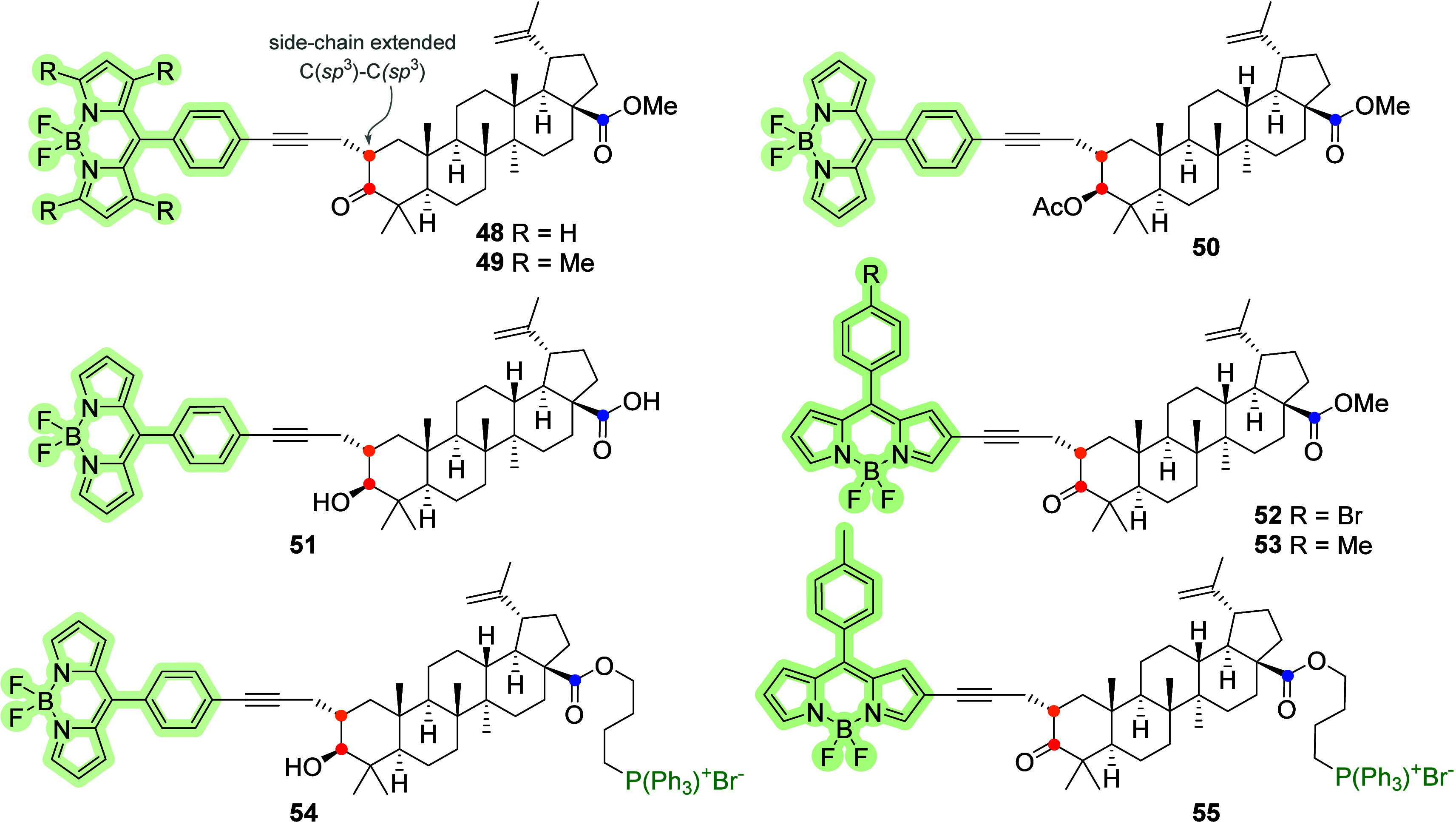
Molecular structures of BA and BA–BODIPY **48**–**55**. The probes were prepared from the C-2 (orange dots)-extended motif of the BA ester. The C-2 BODIPY probes were prepared from the alkynylated triterpene by the Sonogashira cross-coupling reaction with differently halogenated BODIPY dyes. Red dots: C-3 position and blue dots: C-28.

Spivak et al.^69^ developed a new approach for the synthesis of triterpenoid acids conjugated with BODIPY. First, they applied C-2 propynyl derivatives of BA as initial substances for conjugation with the BODIPY dye and synthesized BA–BODIPY conjugates **48**–**53**. Later, they reported a method for synthesizing BA–BODIPY derivatives with a terminal mitochondrial-targeting triphenylphosphonium group on the side chain C-28; see the structure of BA–BODIPY **54** and **55** in Figure 15. The developed protocol enabled the preservation of native 3-OH and 28-COOH groups in the triterpene core.^70^ Unfortunately, these compounds were not further biologically evaluated.

**Figure 16:**
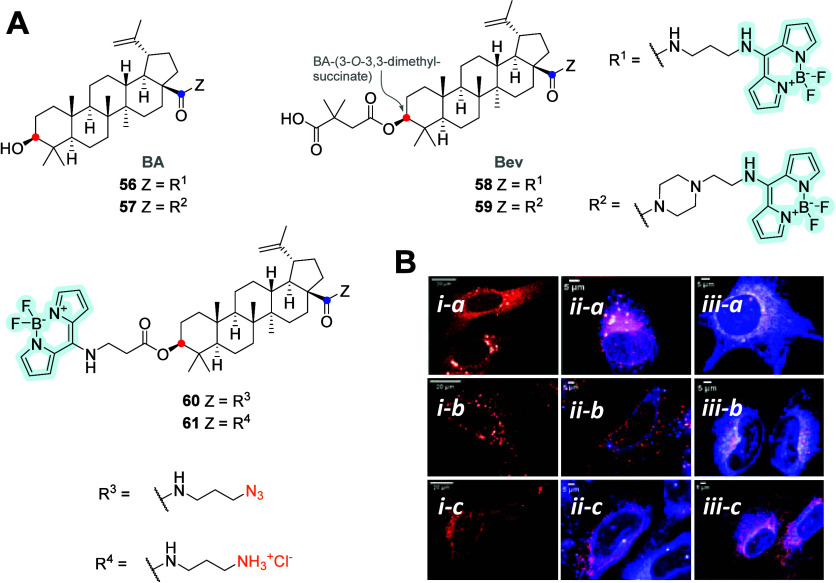
(A) Molecular structures of different blue-emitting triterpene–BODIPY **56**–**61** (BA, betulinic acid; Bev, bevirimat) conjugated via an amide or ester bond with BODIPY (red dots: C-3 position; blue dots: C-28)). (B) Live-cell localization of the cytotoxic probes in U2OS cells. U2OS ER cell lines (*i*-*a*), U2OS GA cell line (*i*-*b*), and U2OS mitochondria cell lines (*i*-*c*) transduced with lentiviral particles expressing tag mCherry targeted to specific subcellular locations. (*ii*-*a*–*c*) Covisualization of probe **56** (blue) with the dye-transfected organelles (red) and (*iii*-*a*–*c*) colabeling of probe **61** (blue).^71^ Copyright 2021, Kodr et al., Licensee MDPI, Basel, Switzerland.

The most extensive results were obtained in a study on BA decorated with polar groups and BODIPY with blue fluorescence. This dye is a unique example in the literature and is suitable for fluorescence applications. Compared with the commonly used coumarins, it exhibits superior photochemical properties, and with the classic BODIPY-FL, it can preserve the natural properties of the conjugates. The BODIPY-labeled analogs derived from BA (**56**–**61**) were prepared by Kodr et al.,^71^ and the structures are shown in Figure 16A. The introduction of an amino moiety into a BA molecule was supposed to enhance its antitumor activity.^72^ The conjugates were tested for cytotoxicity and their influence on the cell cycle, anti-HIV-1 activity, and cellular uptake. Conjugates **56** and **61** were detected in living cells and colocalized with the ER and mitochondria (Figure 16B). Triterpene–BODIPY **56** has a fluorophore attached to the carboxyl group at the C-28 position. Triterpene–BODIPY **58** contains a conjugated amine at the C-28 position. The fluorophore is attached to the hydroxy group at the C-3 position of BA. Because the localization of both compounds was similar, the target may have remained unchanged. However, the free amine groups in the molecule enhance the effect of conjugate **61**.^71^

Derived from *Quillaja saponaria*, QS-21 is a natural product extract exhibiting robust immune-stimulating properties^73^ that were closely explored and led to the approval of two vaccines.^74^ This semipurified extract comprises a blend of triterpene saponins consisting of two principal constituents, QS-21-apiose and QS-21-xylose.^75,76^ These constituents of QS-21 play a critical role in immunostimulation, each characterized by four distinct structural domains: the central triterpene (quillaic acid), a branched trisaccharide located at the C-3 position of the triterpene, a linear tetrasaccharide linked to the C-28 position of the triterpene, and a branched acyl chain tethered to the central fucose moiety of the linear tetrasaccharide.

**Figure 17:**
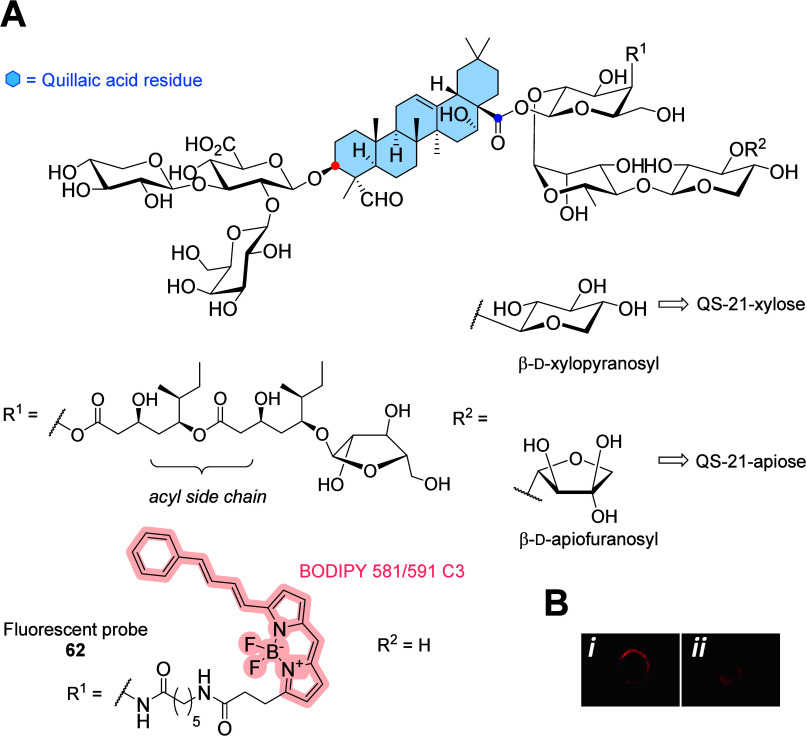
(A) Molecular structures of QS-21 saponin (mixture of QS-21-apiose and QS-21-xylose) and red-emitting saponin–BODIPY conjugate **62** conjugated via an amide bond. (B*-i*) Subcellular localization of fluorescent **62** in immature dendritic cells. (B*-ii*) BODIPY–glycine methyl ester was used as a control.^77^ Copyright 2012, American Chemical Society.

Despite its potential, QS-21 has certain limitations, including low isolation yield, inconsistent composition, lack of purity, and spontaneous hydrolysis of the acyl domain. Therefore, Chea et al.^77^ conducted a study to identify specific substructures within the saponin adjuvant, which is crucial for its adjuvant activity to enable a rational approach for the synthesis of derivatives. They successfully developed a synthetic *Quillaja* scaffold that accommodated the attachment of fluorescent reporter groups while retaining adjuvant activity. This opens the possibility of studying the mechanism of action of QS-21 and other saponin adjuvants. Although saponin–BODIPY **62** exhibited limited immune-potentiating effects and was localized exclusively to the plasma membrane of the examined immature dendritic cells (see Figure 17 for structural and fluorescent images), this synthesis strategy can be used to attach other tags, facilitating inquiries into biodistribution and molecular targets in the future.

## BODIPY-Tagged Probes Inspired by Naturally Active Terpene-Containing Compounds

**Figure 18:**
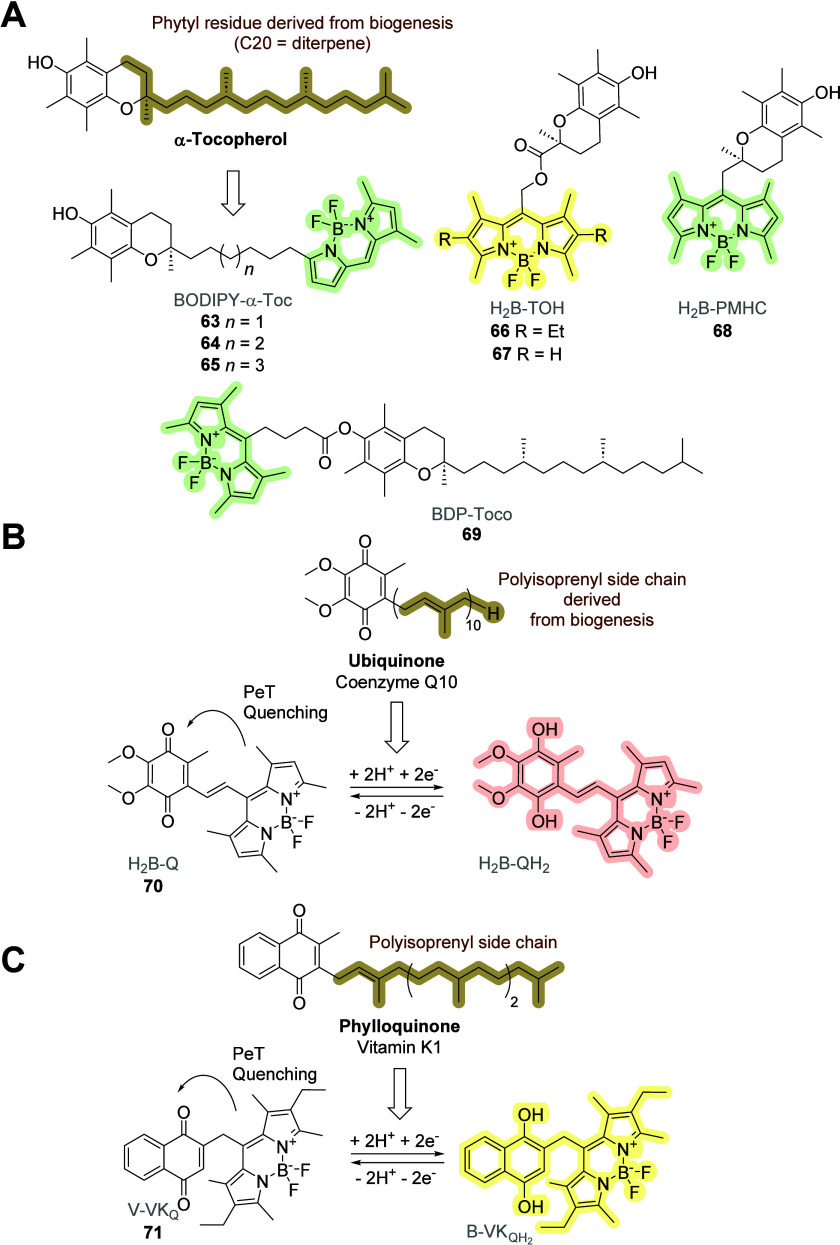
Molecular structures of BODIPY-based probes inspired by (A) tocopherol **63**–**69** (vitamin E), (B) ubiquinone **70** (coenzyme Q; vitamin Q), and (C) phylloquinone **71** (vitamin K1).

The chemical structures of vital vitamins with a terpenoid side chain, exhibiting an oxidation–reduction function in living tissues, have inspired chemists recently to design and synthesize new fluorescent sensors based on their structures (Figure 18). Because BODIPYs are suitable fluorophores owing to their lipophilicity, several studies have focused on this type of compound. Derivatives of vitamin E (tocopherol; Figure 18A), coenzyme Q10 (ubiquinone; Figure 18B), which is now commonly referred to as vitamin Q, and vitamin K1 (phylloquinone; Figure 18C) have been described previously.

Tocopherol derivatives **63**–**65** (Figure 18A) were prepared by Grubbs metathesis of the respective terminal alkenes and subsequent double-bond reduction.^78^ Analog **65**, which contains an eight-carbon chain between the aromatic chromanol and BODIPY, had the highest affinity for α-tocopherol transfer protein (*K*_d_ = 94 ± 3 nM). Because it could not be displaced by cholesterol, the binding was specific, and this derivative exhibited the highest similarity to natural tocopherol.

BODIPY-based probes **66**–**68** were prepared from Trolox.^79,80^ Sensors **66** and **67** contain an ester linkage between the fluorophore and the redox moiety. Derivative **66** exhibited antioxidant properties and exhibited a considerable decrease in the emission quantum yield, which increased considerably upon exposure to peroxyl or alkyl radicals. This result indicates the exhaustion of antioxidants and the onset of radical-controlled oxidation via increased emissions. Derivative **68** was prepared from Trolox by chain extension with one carbon atom via a nitrile intermediate and its subsequent hydrolysis to a carboxylic acid. BODIPY was incorporated into this functional group. The sensor contains a nonhydrolyzable methylene linker between the redox moiety and fluorescent reporter. Lipophilic peroxyl radicals were scavenged with compound **68** with the same efficiency as compound **67**. In general, the substitution of a long terpenoid chain for a short connecting bridge with the BODIPY reporter did not affect the oxidation–reduction ability of the vitamin in the tested models.

The ester conjugate tocopherol–BODIPY **69** was used to form a nanoemulsion, functioning as a lipid anchor.^81^ The derivative exhibited a high molar extinction coefficient, quantum yield, and solubility in oils. Brightly fluorescent nanoemulsions were obtained by spontaneous emulsification, with sizes ranging from 40 to 75 nm and a good polydispersity index. Tocopherol can be generalized as an effective natural lipid anchor for efficiently loading nanoemulsions with fluorophores.

Derivatives **70**(82) and **71**(83) represent other examples of the use of the redox part of vitamins that originally contained terpenoid side chains (Figure 18B and C). The principle of photoinduced electron transfer (PeT), PeT-quenched vs PeT-out state, considerably affects the response of the sensor in terms of emitted radiation. As both sensors exhibited conserved redox properties, they can be used to study and better understand the functions of these vitamins in living systems.

## Conclusions

Herein, BODIPY conjugates developed using various terpenes and terpenoids have been thoroughly discussed. All studies included both chemical and biological evaluations of experimental compounds, each presenting varying levels of detail, such as the use of different microscopic techniques. In summary, fluorescent probes, particularly those based on BODIPY, have not only facilitated terpene-based research but also indicated potential broader applications in natural product research, enabling a deeper understanding of the mechanisms of these compounds. The biological properties, which are highly dependent on the linker used in the conjugate structure and on the properties of the parent-targeting structure, offer insights that can be extrapolated to other classes of natural products. Therefore, future studies should consider the previously reported outcomes. Studies on monoterpenoid-conjugated BODIPY showed their potential for diagnostic applications; these conjugates could label distinct cells without cytotoxic effects. Moreover, sesquiterpenes have shown promising results for future therapeutic applications, particularly in the field of oncology. The Diterpenes and Diterpenoids section highlights the diverse applications of BODIPY-labeled terpenes, taking advantage of their natural binding partners in receptor localization to direct anticancer effects when used as paclitaxel-derived theranostic conjugates. Studies on triterpene carboxylic acids have revealed that the most promising fluorescent probes are derivatives of BA. Other triterpene carboxylic acids also can be conjugated with BODIPY dyes, with some showing potent cytotoxicity in tumor and nontumor cell lines. The best results were obtained by labeling the triterpene molecule at C-3 or C-28, thus keeping the allyl functional group of BA free for binding. Introducing an amino group into the parent structure or linker part was very efficient in terms of promoting biological activity. BODIPY conjugates with vitamin-based terpene structures have proven to be valuable tools for studying the oxidation–reduction function of vitamins, enhancing our understanding of their roles in living systems.

In conclusion, BODIPY terpenes and terpenoid conjugates provide evidence of the synergistic potential of organic chemistry and molecular biology. As we continue to explore their possibilities and limitations, these molecular tools are poised to contribute considerably to our understanding of cellular processes and the development of novel therapeutic agents. The implications of this research extend beyond the current scope of terpenoid-focused research and promise exciting developments in the broader field of natural product studies.

## References

[ref1] Pan S. J.; Zhang H. L.; Wang C. Y.; Yao S. C. L.; Yao S. Q. Target identification of natural products and bioactive compounds using affinity-based probes. Nat. Prod. Rep. 2016, 33 (5), 612–620. 10.1039/C5NP00101C. 26580476

[ref2] Fetz V.; Prochnow H.; Bronstrup M.; Sasse F. Target identification by image analysis. Nat. Prod. Rep. 2016, 33 (5), 655–667. 10.1039/C5NP00113G. 26777141

[ref3] Zhang X. J.; Wen J. Y.; Bidasee K. R.; Besch H. R.; Wojcikiewicz R. J. H.; Lee B.; Rubin R. P. Ryanodine and inositol trisphosphate receptors are differentially distributed and expressed in rat parotid gland. Biochem. J. 1999, 363 (Pt 3), 519–527. 10.1042/bj3400519. PMC1220280 10333498

[ref4] Emmerson P. J.; Archer S.; El-Hamouly W.; Mansour A.; Akil H.; Medzihradsky F. Synthesis and characterization of 4,4-difluoro-4-bora-3a,4a-diaza-s-indacene (BODIPY)-labeled fluorescent ligands for the mu opioid receptor. Biochem. Pharmacol. 1997, 54 (12), 1315–1322. 10.1016/S0006-2952(97)00374-2. 9393674

[ref5] Xu S. T.; Luo S. S.; Yao H.; Cai H.; Miao X. M.; Wu F.; Yang D. H.; Wu X. M.; Xie W. J.; Yao H. Q.; et al. Probing the anticancer action of oridonin with fluorescent analogues: Visualizing subcellular localization to mitochondria. J. Med. Chem. 2016, 59 (10), 5022–5034. 10.1021/acs.jmedchem.6b00408. 27089099

[ref6] Umezawa K.; Yoshida M.; Kamiya M.; Yamasoba T.; Urano Y. Rational design of reversible fluorescent probes for live-cell imaging and quantification of fast glutathione dynamics. Nat. Chem. 2017, 9 (3), 279–286. 10.1038/nchem.2648. 28221345

[ref7] Zhang X.; Ba Q.; Gu Z. N.; Guo D. L.; Zhou Y.; Xu Y. E.; Wang H.; Ye D. J.; Liu H. Fluorescent coumarin-artemisinin conjugates as mitochondria-targeting theranostic probes for enhanced anticancer activities. Chem.—Eur. J. 2015, 21 (48), 17415–17421. 10.1002/chem.201502543. 26458147

[ref8] Zhou X.; Chen X. B.; Du Z. H.; Zhang Y.; Zhang W. J.; Kong X. R.; Thelen J. J.; Chen C. S.; Chen M. J. Terpenoid esters are the major constituents from leaf lipid droplets of camellia sinensis. Front. Plant Sci. 2019, 10, 179 10.3389/fpls.2019.00179. 30863415 PMC6399487

[ref9] Takahashi M.; Kawamura A.; Kato N.; Nishi T.; Hamachi I.; Ohkanda J. Phosphopeptide-dependent labeling of 14–3-3 ζ proteins by fusicoccin-based fluorescent probes. Angew. Chem., Int. Ed. 2012, 51 (2), 509–512. 10.1002/anie.201106995. 22105970

[ref10] Antina E.; Bumagina N.; Marfin Y.; Guseva G.; Nikitina L.; Sbytov D.; Telegin F. BODIPY conjugates as functional compounds for medical diagnostics and treatment. Molecules 2022, 27 (4), 1396 10.3390/molecules27041396. 35209191 PMC8877204

[ref11] Loudet A.; Burgess K. BODIPY dyes and their derivatives: Syntheses and spectroscopic properties. Chem. Rev. 2007, 107 (11), 4891–4932. 10.1021/cr078381n. 17924696

[ref12] Tholl D. Terpene synthases and the regulation, diversity and biological roles of terpene metabolism. Curr. Opin. Plant Biol. 2006, 9 (3), 297–304. 10.1016/j.pbi.2006.03.014. 16600670

[ref13] Juang Y. P.; Liang P. H. Biological and pharmacological effects of synthetic saponins. Molecules 2020, 25 (21), 4974 10.3390/molecules25214974. 33121124 PMC7663351

[ref14] Gang F. L.; Zhu F.; Yang C. F.; Li X. T.; Yang H.; Sun M. X.; Wu W. J.; Zhang J. W. Antifungal, anti-inflamatory and neuritogenic activity of newly-isolated compounds from Disporopsis aspersa. Nat. Prod. Res. 2020, 34 (11), 1521–1527. 10.1080/14786419.2018.1519709. 30445866

[ref15] Ighachane H.; Boualy B.; Ali M. A.; Sedra M. H.; El Firdoussi L.; Lazrek H. B. Catalytic synthesis and antifungal activity of new polychlorinated natural terpenes. Adv. Materi. Sci. Eng. 2017, 2017, 1 10.1155/2017/2784303.

[ref16] Gur’eva Y. A.; Zalevskaya O. A.; Shevchenko O. G.; Slepukhin P. A.; Makarov V. A.; Kuchin A. V. Copper(II) complexes with terpene derivatives of ethylenediamine: synthesis, and antibacterial, antifungal and antioxidant activity. RSC Adv. 2022, 12 (15), 8841–8851. 10.1039/D2RA00223J. 35424859 PMC8985105

[ref17] Novotna E.; Waisser K.; Kunes J.; Palat K.; Buchta V.; Stolarikova J.; Beckert R.; Wsol V. Synthesis and biological activity of quaternary ammonium salt-type agents containing cholesterol and terpenes. Arch. Pharm. 2014, 347 (6), 381–386. 10.1002/ardp.201300407. 24664885

[ref18] Wu H. F.; Morris-Natschke S. L.; Xu X. D.; Yang M. H.; Cheng Y. Y.; Yu S. S.; Lee K. H. Recent advances in natural anti-HIV triterpenoids and analogs. Med. Res. Rev. 2020, 40 (6), 2339–2385. 10.1002/med.21708. 32666531 PMC7554103

[ref19] Song J. G.; Su J. C.; Song Q. Y.; Huang R. L.; Tang W.; Hu L. J.; Huang X. J.; Jiang R. W.; Li Y. L.; Ye W. C.; et al. Cleistocaltones A and B, antiviral phloroglucinol-terpenoid adducts from Cleistocalyx operculatus. Org. Lett. 2019, 21 (23), 9579–9583. 10.1021/acs.orglett.9b03743. 31755722

[ref20] Alho D. P. S.; Salvador J. A. R.; Cascante M.; Marin S. Synthesis and antiproliferative activity of novel A-ring cleaved glycyrrhetinic acid derivatives. Molecules 2019, 24 (16), 2938 10.3390/molecules24162938. 31416117 PMC6721064

[ref21] Kowada T.; Maeda H.; Kikuchi K. BODIPY-based probes for the fluorescence imaging of biomolecules in living cells. Chem. Soc. Rev. 2015, 44 (14), 4953–4972. 10.1039/C5CS00030K. 25801415

[ref22] Guseva G. B.; Antina E. V.; Berezin M. B.; Pavelyev R. S.; Kayumov A. R.; Sharafutdinov I. S.; Lodochnikova O. A.; Islamov D. R.; Usachev K. S.; Boichuk S. V.; et al. Meso-substituted-BODIPY based fluorescent biomarker: Spectral characteristics, photostability and possibilities for practical application. J. Photochem. Photobiol., A 2020, 401, 112783 10.1016/j.jphotochem.2020.112783.

[ref23] Guseva G. B.; Antina E. V.; Berezin M. B.; Pavelyev R. S.; Kayumov A. R.; Ostolopovskaya O. V.; Gilfanov I. R.; Frolova L. L.; Kutchin A. V.; Akhverdiev R. F.; et al. Design, spectral characteristics, and possibilities for practical application of BODIPY FL-labeled monoterpenoid. ACS Appl. Bio Mater. 2021, 4 (8), 6227–6235. 10.1021/acsabm.1c00550. 35006906

[ref24] Guseva G. B.; Antina E. V.; Berezin M. B.; Nikitina L. E.; Gilfanov I. R.; Pavelyev R. S.; Lisovskaya S. A.; Frolova L. L.; Ostolopovskaya O. V.; Rakhmatullin I. Z.; et al. Novel BODIPY conjugates with myrtenol: Design, spectral characteristics, and possibilities for practical application. Inorganics 2023, 11 (6), 241 10.3390/inorganics11060241.

[ref25] Guseva G. B.; Antina E. V.; Berezin M. B.; Smirnova A. S.; Pavelyev R. S.; Gilfanov I. R.; Shevchenko O. G.; Pestova S. V.; Izmest’ev E. S.; Rubtsova S. A.; et al. Design, spectral characteristics, photostability, and possibilities for practical application of BODIPY FL-labeled thioterpenoid. Bioengineering 2022, 9 (5), 210 10.3390/bioengineering9050210. 35621488 PMC9138141

[ref26] Guseva G. B.; Antina E. V.; Berezin M. B.; Ksenofontov A. A.; Bocharov P. S.; Smirnova A. S.; Pavelyev R. S.; Gilfanov I. R.; Pestova S. V.; Izmest’ev E. S.; et al. Conjugate of meso-carboxysubstituted-BODIPY with thioterpenoid as an effective fluorescent probe: Synthesis, structure, spectral characteristics, and molecular docking. Spectrochim. Acta A Mol. Biomol. Spectrosc. 2022, 268, 120638 10.1016/j.saa.2021.120638. 34840052

[ref27] Silva R. O.; Salvadori M. S.; Sousa F. B. M.; Santos M. S.; Carvalho N. S.; Sousa D. P.; Gomes B. S.; Oliveira F. A.; Barbosa A. L. R.; Freitas R. M.; et al. Evaluation of the anti-inflammatory and antinociceptive effects of myrtenol, a plant-derived monoterpene alcohol, in mice. Flavour Fragr. J. 2014, 29 (3), 184–192. 10.1002/ffj.3195.

[ref28] Nikitina L. E.; Startseva V. A.; Dorofeeva L. Y.; Artemova N. P.; Kuznetsov I. V.; Lisovskaya S. A.; Glushko N. P. Antifungal activity of bicyclic monoterpenoids and terpenesulfides. Chem. Nat. Compd. 2010, 46 (1), 28–32. 10.1007/s10600-010-9517-5.

[ref29] Kumar S.; Srivastava S. Establishment of artemisinin combination therapy as first line treatment for combating malaria: Artemisia annua cultivation in India needed for providing sustainable supply chain of artemisinin. Curr. Sci. 2005, 89 (7), 1097–1102.

[ref30] Nazari Z. E.; Iranshahi M. Biologically active sesquiterpene coumarins from Ferula species. Phytother. Res. 2011, 25 (3), 315–323. 10.1002/ptr.3311. 21031633

[ref31] Skytte D. M.; Möller J. V.; Liu H. Z.; Nielsen H. O.; Svenningsen L. E.; Jensen C. M.; Olsen C. E.; Christensen S. B. Elucidation of the topography of the thapsigargin binding site in the sarco-endoplasmic calcium ATPase. Bioorg. Med. Chem. 2010, 18 (15), 5634–5646. 10.1016/j.bmc.2010.06.032. 20615710

[ref32] Sagara Y.; Fernandez-Belda F.; de Meis L.; Inesi G. Characterization of the inhibition of intracellular Ca2+ transport ATPases by thapsigargin. J. Biol. Chem. 1992, 267 (18), 12606–12613. 10.1016/S0021-9258(18)42320-4. 1535623

[ref33] Abrenica B.; Gilchrist J. S. C. Nucleoplasmic Ca2+ loading is regulated by mobilization of perinuclear Ca2+. Cell Calcium 2000, 28 (2), 127–136. 10.1054/ceca.2000.0137. 10970769

[ref34] Vangheluwe P.; Louch W. E.; Ver Heyen M.; Sipido K.; Raeymaekers L.; Wuytack F. Ca2+ transport ATPase isoforms SERCA2a and SERCA2b are targeted to the same sites in the murine heart. Cell Calcium 2003, 34 (6), 457–464. 10.1016/S0143-4160(03)00126-X. 14572804

[ref35] Abrenica B.; Pierce G. N.; Gilchrist J. S. C. Nucleoplasmic calcium regulation in rabbit aortic vascular smooth muscle cells. Can. J. Physiol. Pharmacol. 2003, 81 (3), 301–310. 10.1139/y03-005. 12733828

[ref36] Perez-Gordones M. C.; Serrano M. L.; Rojas H.; Martinez J. C.; Uzcanga G.; Mendoza M. Presence of a thapsigargin-sensitive calcium pump in Trypanosoma evansi: Immunological, physiological, molecular and structural evidences. Exp. Parasitol. 2015, 159, 107–117. 10.1016/j.exppara.2015.08.017. 26297682

[ref37] Jurášek M.; Rimpelová S.; Kmoníčková E.; Drašar P.; Ruml T. Tailor-made fluorescent trilobolide to study its biological relevance. J. Med. Chem. 2014, 57 (19), 7947–7954. 10.1021/jm500690j. 25197766

[ref38] Škorpilová L.; Rimpelová S.; Jurášek M.; Buděšínský M.; Lokajová J.; Effenberg R.; Slepička P.; Ruml T.; Kmoníčková E.; Drašar P. B.; et al. BODIPY-based fluorescent liposomes with sesquiterpene lactone trilobolide. Beilstein J. Org. Chem. 2017, 13, 1316–1324. 10.3762/bjoc.13.128. 28781697 PMC5530629

[ref39] Liu C. Y.; Zhang H. M.; Christofi F. L. Adenylyl cyclase co-distribution with the CaBPs, calbindin-D-28 and calretinin, varies with cell type: assessment with the fluorescent dye, BODIPY forskolin, in enteric ganglia. Cell Tissue Res. 1998, 293 (1), 57–73. 10.1007/s004410051098. 9634598

[ref40] Van Petegem F. Ryanodine receptors: Structure and function. J. Biol. Chem. 2012, 287 (38), 31624–31632. 10.1074/jbc.R112.349068. 22822064 PMC3442496

[ref41] Saldana C.; Diaz-Munoz M.; Antaramian A.; Gonzalez-Gallardo A.; Garcia-Solis P.; Morales-Tlalpan V. MCF-7 breast carcinoma cells express ryanodine receptor type 1: functional characterization and subcellular localization. Mol. Cell. Biochem. 2009, 323 (1–2), 39–47. 10.1007/s11010-008-9962-7. 19082546

[ref42] Braun D. C.; Cao Y. Y.; Wang S. M.; Garfield S. H.; Hur G. M.; Blumberg P. M. Role of phorbol ester localization in determining protein kinase C or RasGRP3 translocation: Real-time analysis using fluorescent ligands and proteins. Mol. Cancer Ther. 2005, 4 (1), 141–150. 10.1158/1535-7163.141.4.1. 15657361

[ref43] Czikora A.; Lundberg D. J.; Abramovitz A.; Lewin N. E.; Kedei N.; Peach M. L.; Zhou X. L.; Merritt R. C.; Craft E. A.; Braun D. C.; et al. Structural Basis for the Failure of the C1 Domain of Ras Guanine Nucleotide Releasing Protein 2 (RasGRP2) to Bind Phorbol Ester with High Affinity. J. Biol. Chem. 2016, 291 (21), 11133–11147. 10.1074/jbc.M116.725333. 27022025 PMC4900263

[ref44] Ikezoe T.; Chen S. S.; Tong X. J.; Heber D.; Taguchi H.; Koeffler H. P. Oridonin induces growth inhibition and apoptosis of a variety of human cancer cells. Int. J. Oncol. 2003, 23 (4), 1187–1193. 10.3892/ijo.23.4.1187. 12964003

[ref45] Hu H. Z.; Yang Y. B.; Xu X. D.; Shen H. W.; Shu Y. M.; Ren Z.; Li X. M.; Shen H. M.; Zeng H. T. Oridonin induces apoptosis via PI3K/Akt pathway in cervical carcinoma HeLa cell line. Acta Pharmacol. Sin. 2007, 28 (11), 1819–1826. 10.1111/j.1745-7254.2007.00667.x. 17959034

[ref46] He H. B.; Jiang H.; Chen Y.; Ye J.; Wang A. L.; Wang C.; Liu Q. S.; Liang G. L.; Deng X. M.; Jiang W.; et al. Oridonin is a covalent NLRP3 inhibitor with strong anti-inflammasome activity. Nat. Commun. 2018, 9 (1), 2550 10.1038/s41467-018-04947-6. 29959312 PMC6026158

[ref47] Vasaturo M.; Cotugno R.; Fiengo L.; Vinegoni C.; Dal Piaz F.; De Tommasi N. The anti-tumor diterpene oridonin is a direct inhibitor of Nucleolin in cancer cells. Sci. Rep. 2018, 8 (1), 16735 10.1038/s41598-018-35088-x. 30425290 PMC6233161

[ref48] Huang J. L.; Yan X. L.; Li W.; Fan R. Z.; Li S.; Chen J. H.; Zhang Z. H.; Sang J.; Gan L.; Tang G. H.; et al. Discovery of highly potent daphnane diterpenoids uncovers importin-beta 1 as a druggable vulnerability in castration-resistant prostate cancer. J. Am. Chem. Soc. 2022, 144 (38), 17522–17532. 10.1021/jacs.2c06449. 36103720

[ref49] Dehelean C. A.; Marcovici I.; Soica C.; Mioc M.; Coricovac D.; Iurciuc S.; Cretu O. M.; Pinzaru I. Plant-Derived Anticancer Compounds as New Perspectives in Drug Discovery and Alternative Therapy. Molecules 2021, 26 ( (4), ), 1109 10.3390/molecules26041109. 33669817 PMC7922180

[ref50] Kellogg E. H.; Hejab N. M. A.; Howes S.; Northcote P.; Miller J. H.; Diaz J. F.; Downing K. H.; Nogales E. Insights into the distinct mechanisms of action of taxane and non-taxane microtubule stabilizers from Cryo-EM structures. J. Mol. Biol. 2017, 429 (5), 633–646. 10.1016/j.jmb.2017.01.001. 28104363 PMC5325780

[ref51] Mitchell M. J.; Billingsley M. M.; Haley R. M.; Wechsler M. E.; Peppas N. A.; Langer R. Engineering precision nanoparticles for drug delivery. Nat. Rev. Drug Discovery 2021, 20 (2), 101–124. 10.1038/s41573-020-0090-8. 33277608 PMC7717100

[ref52] Sun T. T.; Lin W. H.; Zhang W.; Xie Z. G. Self-assembly of amphiphilic drug-dye conjugates into nanoparticles for imaging and chemotherapy. Chem.—Asian J. 2016, 11 (22), 3174–3177. 10.1002/asia.201601206. 27652521

[ref53] Zhang T.; Zhang W.; Zheng M.; Xie Z. G. Near-infrared BODIPY-paclitaxel conjugates assembling organic nanoparticles for chemotherapy and bioimaging. J. Colloid Interface Sci. 2018, 514, 584–591. 10.1016/j.jcis.2017.12.074. 29294445

[ref54] Xia J. X.; Pei Q.; Zheng M.; Xie Z. G. An activatable fluorescent prodrug of paclitaxel and BODIPY. J. Mater. Chem. B 2021, 9 (9), 2308–2313. 10.1039/D0TB02510K. 33616144

[ref55] Wijesooriya C. S.; Peterson J. A.; Shrestha P.; Gehrmann E. J.; Winter A. H.; Smith E. A. A photoactivatable BODIPY Probe for localization-based super-resolution cellular imaging. Angew. Chem., Int. Ed. 2018, 57 (39), 12685–12689. 10.1002/anie.201805827. 30247796

[ref56] Rogers D.; Phillips F. L.; Joshi B. S.; Viswanathan N. Revised structures of the triterpenes Q, T, and U from Salacia prinoides DC; X-ray crystal structure of triterpene T. J. Chem. Soc., Chem. Commun. 1980, (22), 1048–1049. 10.1039/c39800001048.

[ref57] Vo N. N. Q.; Nomura Y.; Muranaka T.; Fukushima E. O. Structure-activity relationships of pentacyclic triterpenoids as inhibitors of cyclooxygenase and lipoxygenase enzymes. J. Nat. Prod. 2019, 82 (12), 3311–3320. 10.1021/acs.jnatprod.9b00538. 31774676

[ref58] Villarroel-Vicente C.; Gutierrez-Palomo S.; Ferri J.; Cortes D.; Cabedo N. Natural products and analogs as preventive agents for metabolic syndrome via peroxisome proliferator-activated receptors: An overview. Eur. J. Med. Chem. 2021, 221, 113535 10.1016/j.ejmech.2021.113535. 33992930

[ref59] Yang H. J.; Dou Q. P. Targeting apoptosis pathway with natural terpenoids: Implications for treatment of breast and prostate cancer. Curr. Drug Targets 2010, 11 (6), 733–744. 10.2174/138945010791170842. 20298150 PMC3306610

[ref60] Rybalkina E. Y.; Moiseeva N. I.; Karamysheva A. F.; Eroshenko D. V.; Konysheva A. V.; Nazarov A. V.; Grishko V. V. Triterpenoids with modified A-ring as modulators of P-gp-dependent drug-resistance in cancer cells. Chem. Biol. Interact. 2021, 348, 109645 10.1016/j.cbi.2021.109645. 34516973

[ref61] Fulda S.; Friesen C.; Los M.; Scaffidi C.; Mier W.; Benedict M.; Nunez G.; Krammer P. H.; Peter M. E.; Debatin K. M. Betulinic acid triggers CD95 (APO-1/Fas)- and p53-independent apoptosis via activation of caspases in neuroectodermal tumors. Cancer Res. 1997, 57 (21), 4956–4964. 9354463

[ref62] Xu Y.; Shu B.; Tian Y.; Wang G. X.; Wang Y. J.; Wang J. W.; Dong Y. F. Oleanolic acid induces osteosarcoma cell apoptosis by inhibition of Notch signaling. Mol. Carcinog. 2018, 57 (7), 896–902. 10.1002/mc.22810. 29566282

[ref63] Bhola P. D.; Letai A. Mitochondria-judges and executioners of cell death sentences. Mol. Cell 2016, 61 (5), 695–704. 10.1016/j.molcel.2016.02.019. 26942674 PMC4806554

[ref64] Krajcovicova S.; Stankova J.; Dzubak P.; Hajduch M.; Soural M.; Urban M. A Synthetic approach for the rapid preparation of BODIPY conjugates and their use in imaging of cellular drug uptake and distribution. Chem.—Eur. J. 2018, 24 (19), 4957–4966. 10.1002/chem.201706093. 29411907

[ref65] Dubinin M. V.; Semenova A. A.; Ilzorkina A. I.; Penkov N. V.; Nedopekina D. A.; Sharapov V. A.; Khoroshavina E. I.; Davletshin E. V.; Belosludtseva N. V.; Spivak A. Y.; et al. Mitochondria-targeted prooxidant effects of betulinic acid conjugated with delocalized lipophilic cation F16. Free Radic. Biol. Med. 2021, 168, 55–69. 10.1016/j.freeradbiomed.2021.03.036. 33812008

[ref66] Gu M.; Zhao P.; Zhang S. Y.; Fan S. J.; Yang L.; Tong Q. C.; Ji G.; Huan C. Betulinic acid alleviates endoplasmic reticulum stress-mediated nonalcoholic fatty liver disease through activation of farnesoid X receptors in mice. Br. J. Pharmacol. 2019, 176 (7), 847–863. 10.1111/bph.14570. 30635917 PMC6433649

[ref67] Brandes B.; Hoenke S.; Fischer L.; Csuk R. Design, synthesis and cytotoxicity of BODIPY FL labelled triterpenoids. Eur. J. Med. Chem. 2020, 185, 111858 10.1016/j.ejmech.2019.111858. 31718946

[ref68] Hoenke S.; Serbian I.; Deigner H. P.; Csuk R. Mitocanic di- and triterpenoid rhodamine B conjugates. Molecules 2020, 25 (22), 5443 10.3390/molecules25225443. 33233650 PMC7699795

[ref69] Gubaidullin R.; Nedopekina D.; Tukhbatullin A.; Davletshin E.; Spivak A. Design, synthesis, and photophysical properties of BODIPY-labeled lupane triterpenoids. Chem. Proc. 2021, 3 (1), 11 10.3390/ecsoc-24-08102.

[ref70] Spivak A. Y.; Davletshin E. V.; Gubaidullin R. R.; Tukhbatullin A. A.; Nedopekina D. A. Synthesis of Bodipy-labeled fluorescent betulinic acid derivatives with a terminal triphenylphosphonium group on side-chain C-28. Chem. Nat. Compd. 2022, 58 (6), 1062–1068. 10.1007/s10600-022-03869-6.

[ref71] Kodr D.; Stanková J.; Rumlová M.; Džubák P.; Řehulka J.; Zimmermann T.; Křížová I.; Gurská S.; Hajdúch M.; Drašar P. B.; et al. Betulinic acid decorated with polar groups and blue emitting BODIPY Dye: Synthesis, cytotoxicity, cell-cycle analysis and anti-HIV profiling. Biomedicines 2021, 9 (9), 1104 10.3390/biomedicines9091104. 34572290 PMC8472287

[ref72] Bildziukevich U.; Rarova L.; Janovska L.; Saman D.; Wimmer Z. Enhancing effect of cystamine in its amides with betulinic acid as antimicrobial and antitumor agent in vitro. Steroids 2019, 148, 91–98. 10.1016/j.steroids.2019.04.004. 31022408

[ref73] Kensil C. R.; Patel U.; Lennick M.; Marciani D. Separation and characterization of saponins with adjuvant activity from Quillaja-Saponaria molina cortex. J. Immunol. 1991, 146 (2), 431–437. 10.4049/jimmunol.146.2.431. 1987271

[ref74] Lacaille-Dubois M. A. Updated insights into the mechanism of action and clinical profile of the immunoadjuvant QS-21: A review. Phytomedicine 2019, 60, 152905 10.1016/j.phymed.2019.152905. 31182297 PMC7127804

[ref75] Soltysik S.; Bedore D. A.; Kensil C. R. Adjuvant acitvity of QS-21 isomers. Ann. N.Y. Acad. Sci. 1993, 690, 392–395. 10.1111/j.1749-6632.1993.tb44041.x. 8368766

[ref76] Jacobsen N. E.; Fairbrother W. J.; Kensil C. R.; Lim A.; Wheeler D. A.; Powell M. F. Structure of the saponin adjuvant QS-21 and its base-catalyzed isomerization product by H-1 and natural abundance C-13 NMR spectroscopy. Carbohydr. Res. 1996, 280 (1), 1–14. 10.1016/0008-6215(95)00278-2. 8581890

[ref77] Chea E. K.; Fernandez-Tejada A.; Damani P.; Adams M. M.; Gardner J. R.; Livingston P. O.; Ragupathi G.; Gin D. Y. Synthesis and preclinical evaluation of QS-21 variants leading to simplified vaccine adjuvants and mechanistic probes. J. Am. Chem. Soc. 2012, 134 (32), 13448–13457. 10.1021/ja305121q. 22866694 PMC3436428

[ref78] West R.; Panagabko C.; Atkinson J. Synthesis and characterization of BODIPY-alpha-tocopherol: A fluorescent form of vitamin E. J. Org. Chem. 2010, 75 (9), 2883–2892. 10.1021/jo100095n. 20387845 PMC2891523

[ref79] Oleynik P.; Ishihara Y.; Cosa G. Design and synthesis of a BODIPY-alpha-tocopherol adduct for use as an off/on fluorescent antioxidant indicator. J. Am. Chem. Soc. 2007, 129 (7), 1842–1843. 10.1021/ja066789g. 17253686

[ref80] Krumoya K.; Friedland S.; Cosa G. How lipid unsaturation, peroxyl radical partitioning, and chromanol lipophilic tail affect the antioxidant activity of alpha-tocopherol: Direct visualization via high-throughput fluorescence studies conducted with fluorogenic alpha-tocopherol analogues. J. Am. Chem. Soc. 2012, 134 (24), 10102–10113. 10.1021/ja301680m. 22568598

[ref81] Wang X. Y.; Bou S.; Klymchenko A. S.; Anton N.; Collot M. Ultrabright green-emitting nanoemulsions based on natural lipids-BODIPY conjugates. Nanomaterials 2021, 11 (3), 826 10.3390/nano11030826. 33807096 PMC8005018

[ref82] Greene L. E.; Godin R.; Cosa G. Fluorogenic ubiquinone analogue for monitoring chemical and biological redox processes. J. Am. Chem. Soc. 2016, 138 (35), 11327–11334. 10.1021/jacs.6b06899. 27508986

[ref83] Belzile M. N.; Godin R.; Durantini A. M.; Cosa G. Monitoring chemical and biological electron transfer reactions with a fluorogenic vitamin K analogue probe. J. Am. Chem. Soc. 2016, 138 (50), 16388–16397. 10.1021/jacs.6b09735. 27998090

